# Experimental Method of Emission Generation Calibration Based on Reference Liquids Characterization

**DOI:** 10.3390/ijerph16132262

**Published:** 2019-06-26

**Authors:** Sébastien Soulet, Marie Duquesne, Jean Toutain, Charly Pairaud, Hélène Lalo

**Affiliations:** 1 Laboratoire Français du E-Liquide, 218 avenue du Haut-Levêque, 33600 Pessac, France; 2 Université de Bordeaux, CNRS, I2M Bordeaux, Site ENSAM, Esplanade des Arts et Métiers, F-33405 Talence CEDEX, France; 3 Bordeaux INP, CNRS, I2M Bordeaux, ENSCBP, 16 avenue Pey Berland, 33607 Pessac CEDEX, France

**Keywords:** electronic cigarette, e-liquid composition, emission generation, standardization, E-liquid benchmarking, vaporization, density

## Abstract

This work focuses on an experimental study of the influence of e-liquid composition on the mass of vaporized e-liquid after standardized emission generation using a U-SAV (Universal System for Analysis of Vaping) vaping machine. All the experiments were based on the use of a Cubis 1Ω clearomiser and on the standard protocol for electronic cigarettes emission generation. Currently, there is no standardized method available to calibrate the emission generations of electronic cigarettes. Since the e-liquid compositions are not always known, we propose a simple, practical, effective, and fast method of emission generation calibration. Therefore, this paper examines a major issue in this new and constantly evolving field, allowing the validation of the emission generation results. To our knowledge, this method is a novelty in our discipline and could be easily developed in laboratories. Pure propylene-glycol, glycerol, ethanol, and water and their mixtures (20 e-liquids) were tested as reference materials, allowing an e-liquids benchmarking and the characterization of 800 commercial e-liquids (with known and unknown compositions) at a fixed power and for one inhalation profile (3 s puff duration and 55 mL of puff volume). The influence of ethanol and/or water addition in the e-liquid was characterized.

## 1. Introduction

Many ongoing recent research has focused on liquid vaporization [1,2,3,4] in various applications, such as industrial drying. A recently marketed device is based on the same principle, the electronic cigarette (e-cig). An electrically heated resistive wire vaporizes the liquids for e-cigs (called e-liquids). Thousands of commercial liquids used in e-cig, each with peculiar composition and taste, are currently available in the market [5]. They are mainly composed of propylene-glycol (PG) and vegetable glycerin (VG). These two compounds make the “base” of a liquid to which ethanol (EtOH) and/or water (H_2_O) is added. Finally, aromas in low proportions can also be added, giving, for example, fruity tastes to the inhaled vapor. The vapor–liquid equilibria (VLE) of these pure liquids are well known separately [6], but data focusing on the mixtures are incomplete. Typically, the {EtOH-H_2_O} azeotropic mixture is largely used and studied in petro-chemistry and many papers deal with its VLE [7,8,9,10,11,12]. Few papers have shown results for the {H_2_O-VG} binary system [13] and the {EtOH-H_2_O-VG} ternary one [14,15]. As far as we know, the VLE of the other multi-component systems (binary, ternary, and quaternary) containing PG have not been studied yet. Only two publications using PG were found and were related to the {H_2_O-PG [16] and {EtOH-PG} [17] binary systems. Many recent e-cig studies highlighted the influence of the PG:VG ratio (in volume) on the emission qualities [18,19,20,21,22]. An e-liquid is mostly analyzed as a binary mixture but some other molecules added in e-liquids could also be present in significant concentrations, thus having an impact on the emission. In the studies about e-cigs aiming to characterize the influence of parameters on the generated emission, the mass of vaporized e-liquid (MVE) is a fundamental and key measurement. This value represents the mass lost by the atomizer over the experiments (i.e., the mass of liquid vaporized). The atomizers are weighted filled of liquid before and after the vaporization explaining the mass loss of the atomizers. Hence, our experiments were conducted regarding this physical parameter. The overall objective of this work is twofold. Firstly, it aims at investigating the influence of the e-liquid composition on its vaporization behavior. Secondly, it aims at developing an innovative experimental method to calibrate the emission generation experiments.

This paper focuses on an experimental study of the four main pure compounds of e-liquids (PG, VG, EtOH, H_2_O) and of their mixtures (binary, ternary, and quaternary ones). To study the influence of the composition of an e-liquid on its vaporization behavior and develop an innovative experimental method to calibrate the emission generation experiments, four steps were required. The first one consisted in estimating the MVE of four pure liquids using the Cubis 1Ω clearomiser (Joyetech, Shenzhen, China) in its power range, i.e., from 10 to 25 W. Then, experiments consisted in measuring the density of the previous four pure liquids, of a binary system ({PG-VG}), of two ternary systems ({PG-VG-EtOH} and {PG-VG-H_2_O}), and of a quaternary one ({PG-VG-EtOH-H_2_O}). All of them in different proportions were considered as reference materials (20 liquids in total) and used for e-liquid calibration. This calibration step allowed consideration of all of the previous pure and multi-component systems as reference materials and their MVE were measured at the determined power of reference for emission generation calibration. After this calibration step, a series of experiments consisted in measuring the density of 800 commercial liquids analyzed for Tobacco Product Directive notifications and comparing the obtained results to the calibrated e-liquids. Among these 800 commercial liquids, the compositions of 642 of them were known. The vaporization behavior of the reference and the commercial liquids were investigated and the performances of the proposed experimental method were evaluated and discussed.

## 2. Materials and Methods

### 2.1. Reference E-Liquids

All of the 800 commercial tested e-liquids mainly contain two compounds in different volume ratios: PG and VG. The manufacturers generally add nicotine and flavors along with H_2_O and/or EtOH. Indeed, H_2_O and EtOH are used as diluents for flavors, and their volume fraction never exceeds 10% in total (either 10% of H_2_O; or 10% of EtOH; or 5% of H_2_O + 5% of EtOH). We performed a first study aiming at only testing pure PG and VG as well as their mixtures. Then, a second one consisted in also considering H_2_O and EtOH. This work reports only on both these first studies. Further works will consist in considering all compounds, even those in low proportions (nicotine, flavors, etc.).

Our method was calibrated using only four pure liquids: PG, VG, EtOH, and H_2_O. To be representative of the 800 commercial e-liquids, we studied a large panel of reference liquids based on the mixtures of the considered four pure liquids in 20 different proportions (5 binary systems, 10 ternary systems, and 5 quaternary systems). This study aimed at being able to identify and highlight common features and behaviors according to e-liquid compositions.

In total, 20 reference e-liquids were manufactured according to 4 ranges. Each range was composed of five binary mixtures made of a base of PG and VG with the volume/volume (*v*/*v*) ratio of 100/0, 80/20, 50/50, 20/80, and 0/100. Then, two ranges of ternary mixtures were made with EtOH or H_2_O added at the volume percent of 10% because in commercial liquids, the ratio of EtOH and H_2_O never exceeds 10% in the volume of the final solution. Finally, the last range was made of quaternary mixtures of the studied pure liquids. EtOH and H_2_O were added at the volume percent of 5% to 5%. General information about the pure liquids tested in this study is given in Table 1.

Their molar mass (M), density (ρ), boiling point (T_b_), heat conductivity (λ), heat capacity (C_p_), and enthalpy of vaporization (H_v_) (given at ambient pressure and temperature) are listed in Table 2.

The 20 reference liquids were made following the same protocol. In each case, the liquid was prepared in a 20 mL e-liquid bottle. Firstly, the VG volume was introduced in the bottle followed by PG then EtOH and H_2_O (if needed). The volumes were pipetted with a 1 mL pipette (precision of 0.008 mL). Each bottle was mixed by hand-shaking during 5 min. Before each experiment, the bottle was re-shaken during 2 min.

A series of measurements was performed to determine the density of the 20 reference liquids. First, the mixture densities were theoretically estimated (ρ_ev_) using the mass fraction of each pure liquid (x_i_) and the density (ρ_i_) of each of the (n)-pure liquids following Equation (1):

(1) $$\rho_{\mathrm{ev}}=\sum_{i}^{n}x_{i}\rho_{i}.$$

We prepared and measured the density (ρ_m_) of the binary, ternary, and quaternary systems by weighting three times a volume of 1 mL of a liquid collected using a pipette with a precision of 0.008 mL. Their densities were measured at ambient pressure and temperature, and then compared with the theoretical ones.

### 2.2. Standard Device and Operating Conditions

#### 2.2.1. Reference Device

**Figure 1:**
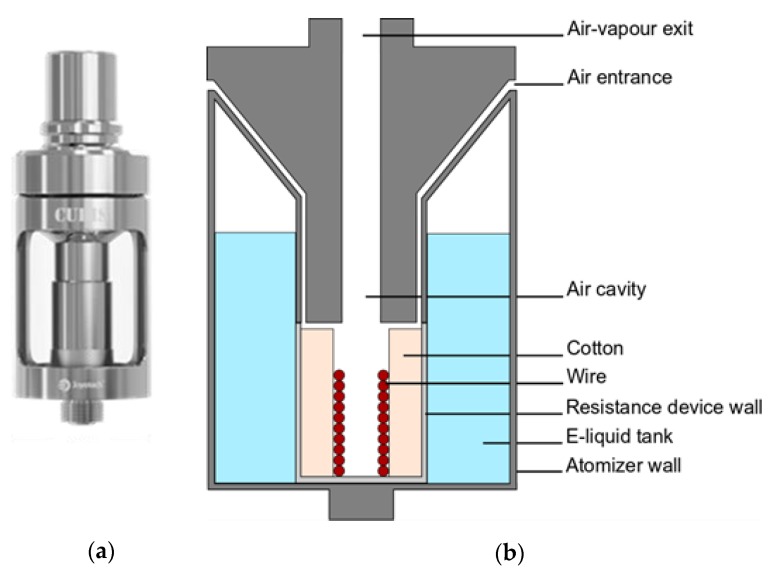
(**a**) Picture of the Cubis clearomiser and (**b**) sectional scheme.

The commercial clearomiser used in this study was a Cubis one (Figure 1a) supplied by Joytech^TM^. It is made of a coil and a wick (organic cotton). The coil is composed of a rolled wire. This wire is composed of three parts: Both extremities are non-resistive (to avoid the burning of the closest elements with which they are in contact with) and the central part, connected to both non-resistive extremities, is resistive to ensure optimal heating of the soaked wick. The wick is rolled around this coil. From the vertical axis to the outer surface of the device, there is first, the air cavity where the inhaled air is mixed with the generated vapor. Then, the heating wire is rolled with a diameter of 2 or 3 mm. The wick is inserted between the central part of the wire and the resistance wall. The e-liquid tank takes place between the resistance wall and the clearomiser wall (Figure 1b).

A Cubis clearomiser is sold with three coils (0.5, 1,and 1.5 Ω), which allows use by beginners as well as experimented e-cig users. A Cubis clearomiser with a 1 Ω coil (see general information in Table 3) was the only one tested and used in this study due to its representative of an intermediate vape behavior.

This device’s repeatability was proven in [23]. Indeed, the results showed that with two standardized liquids [24] (having different PG:VG ratios but the same quantities of other added molecules), the MVE over 36 experiments presented a standard deviation inferior to 8%. The repeatability of the experiments performed with the Cubis was illustrated in [25]. The MVE was measured over 22 series of 20 puffs (without any refilling) and presented a standard deviation lower than 3%.

This device is also used as a reference in the French Laboratory of E-Liquid (LFEL) for the emission analysis required for Tobacco Products Directive (TPD) compliance. In the following experiments, a triplicate of this Cubis clearomiser was tested in order to measure variability over the devices and to allow a reliable evaluation of the influence of the e-liquid composition on vaporization and, therefore, on its MVE.

Its reproducibility and repeatability were already performed for the characterization in [25], and the Cubis clearomiser was used as a reference in this work.

#### 2.2.2. Reference Power

The influence of the power supplied to the Cubis 1Ω coil (presented in Section 2.2.1.) was also characterized in [25]. Three regimes were identified. The optimal regime of the Cubis 1Ω coil ranges from 10 to 25 W and its curve of the MVE versus supplied power over this range is characterized by a linear trend with a slope inversely proportional to the e-liquid enthalpy of vaporization. Besides, this is in accordance with the power range requirement of the manufacturer. However, this observation has been realized with a specific e-liquid. Hence, the optimal regime can be different for other e-liquids.

**Figure 2:**
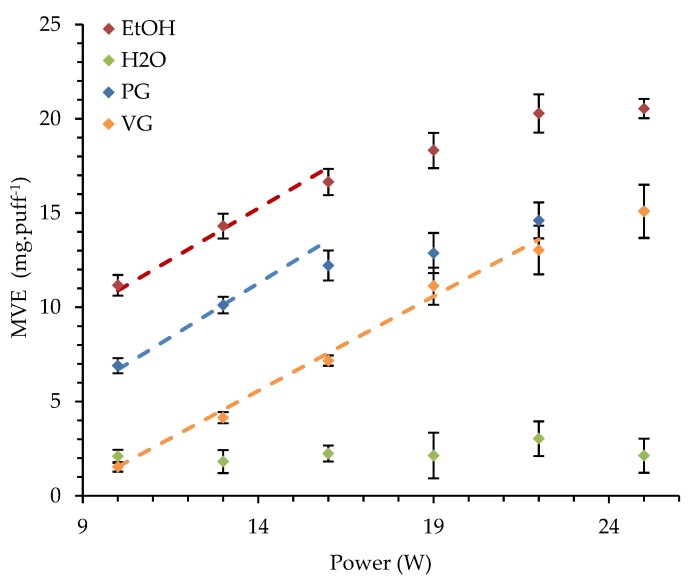
Influence of the supplied power on the mass of vaporized e-liquid (MVE) (dots) of pure liquids (ethanol (EtOH), propylene-glycol (PG), glycerol (VG), and water (H_2_O)) and optimal regimes (in dash line) of EtOH, PG, and VG.

A preliminary series of experiments was performed to determine the optimal power that should be used as a reference power for all the experiments. The aim was to test each pure liquid and to characterize the influence of the power, ranging from 10 to 25 W by intervals of 3 W, on the MVE. Thus, we were able to determine whether the optimal regime is the same for different liquids. Figure 2 presents the influence of the reached supplied power on the MVE for the four studied pure liquids. The optimal EtOH, PG, and VG regimes are also illustrated.

When H_2_O was used, the MVE did not seem to be influenced by the supplied power, revealing that the optimal regime was not reached. However, the average MVE for H_2_O was equal to 2.24 mg·puff^−1^ with a standard deviation of 0.41 mg·puff^−1^. Although the delivered energy increased from 10 W to 25 W, it seems insufficient to reach an optimal regime of vaporization for H_2_O during the puff duration. H_2_O has a heat capacity and an enthalpy of vaporization at least 1.67 times higher (respectively 2.26 times for the enthalpy of vaporization) compared to PG, VG, and EtOH (see Table 2). The vaporization costs more energy for H_2_O. Additionally, H_2_O has a thermal conductivity at least 2 times higher than PG, VG, and EtOH. In other words, H_2_O will diffuse more energy and less energy will be allocated for its vaporization. Finally, H_2_O has a molar mass at least 2.5 times the PG, VG, and EtOH ones. Consequently, when H_2_O is added in e-liquids at a fixed volume concentration (common way to manufacture e-liquids), its molar concentration is higher, leading to a higher thermal and thermodynamical impact. They are good thermo-physical reasons for the H_2_O volume fraction to be added in low proportions in commercial liquids. The three other MVE are influenced by the supplied power. For EtOH and PG, the optimal regime was over at 14 to 16 W and 22 W for VG. At high supplied power, the same mass of PG and VG was vaporized. This observation was also mentioned by Kosminder et al. in [26].

Based on these results, we decided to fix the reference power for this clearomiser at 15 W. Considering the vaporization behavior, this value corresponds to a moderate use of the device. Besides, this power value is also used for TPD notification in the LFEL analytical laboratory.

#### 2.2.3. Vaping Machine

So far, e-cigs have always been tested on smoking machines, adapted to vaping products. The vaping machine Universal System for Analysis of Vaping (U-SAV) [23], the first vaping machine especially designed to test e-cigs, was designed to control all the physical parameters influencing e-liquid vaporization: Power, resistance, flow rate, and inhalation time. U-SAV is a practical tool, ensuring the stability of the required power and avoiding power fluctuations due to battery discharges, which could impact on the quality of the regulation (for more details on U-SAV, see [26,27]). The U-SAV protocol was based on the AFNOR (French Association for Standardization) protocol [24]. U-SAV’s reproducibility and repeatability in the air-flow profile as well as supplied power generation were previously demonstrated in [23].

Additionally, in emission generation for TPD notification (defined as “classic installation”), a cryogenic trap in series with impingers is connected to the drip-tip, allowing the emission to be caught. Since our aim was to characterize the influence of physical parameters on the MVE, a simplified installation (defined as the “specific installation”) was realized without any trap. Firstly, we compared the MVE obtained with both installations (classical and specific ones) using pure PG (reference e-liquid having the lowest density) and pure VG (reference e-liquid having the highest density), representing both extremities of the studied e-liquids’ density range. Although all e-liquids are different, some products’ commercially named liquids “base” are only composed with PG and/or VG. They are sold for the do it yourself (DIY) practice, in order to dilute concentrated flavors with PG and/or VG solutions. They are included in the 800 analyzed commercial e-liquid tested in this work (see Section 2.1). Hence, commercial “pure VG bases” and “pure PG bases” coming from different manufacturers were tested. The MVE obtained with both installations (classic and specific installations) are reported in Table 4. In all cases (VG or PG bases), the relative standard deviations did not exceed 11% (maximum reached for pure VG bases). The corresponding averages and standard deviations over six measurements of the obtained MVE are also reported in Table 4 and show that the trap has no influence on the MVE. Indeed, in TPD conditions (Cubis 1 Ω), the difference was lower than 5%. Therefore, the specific installation allows comparison of the commercial liquids with the 20 reference liquids’ results.

### 2.3. Physical Reference Parameters

Since 2014, LFEL has been equipped with an analytical laboratory, allowing the investigations of e-liquids in both liquid and vapor phases. TPD required the declaration of each vaping products (e-liquid, e-cig, etc.) available in the market as well as the analysis of e-liquids in the vapor phase. Each pure component with a concentration higher than 0.1% in mass must be notified. As an analysis provider, LFEL has already analyzed 800 commercial liquids, which could be separated in two categories. The first one is composed of 642 liquids that were analyzed for TPD compliance, i.e., with known compositions. The second category is composed of 158 e-liquids that were analyzed for emissions composition, i.e., with unknown composition.

**Figure 3:**
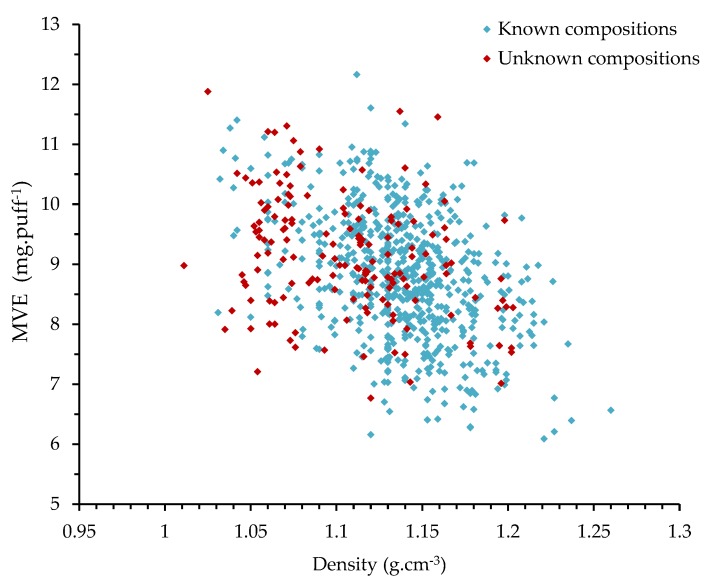
MVE (mass of vaporized e-liquid) of 800 commercial liquids measured after standardized emission generation versus their densities.

Currently, there is no standardized method available to calibrate the emission generation. Since e-liquid compositions are not always known, we propose in this paper a simple, practical, effective, and fast method to calibrate emission generation based on the measurement of the tested e-liquid density and the corresponding MVE. The density was simply measured by weighting 1 mL of e-liquid, collected before the experiments with a micropipette (precision: 0.008 mL). The density measurements of the 800 studied commercial liquids are illustrated in Figure 3.

Some e-liquids can have the same density but different vaporization behaviors characterized by distinct values of MVE (see Figure 3). As an example, 32 commercial liquids have a density of 1.12 g·cm^−3^ and the corresponding MVEs vary from 6.16 mg·puff^−1^ to 11.61 mg·puff^−1^. In this paper, we propose an experimental benchmarking of e-liquids based on the MVE measurement in response to a standardized protocol. The calibration was performed using the main pure liquids used in commercial e-liquids and their mixtures as reference liquids in order to be able to validate the emission generation of all e-liquids.

### 2.4. Systematic Protocols and Reproducibility of the Performed Measurements

In agreement with the International Organization for Standardization (ISO) standard [27], each puff had a period of 30 s, including 3 s of vaporization and 27 s of rest. In this study, the air flow rate was programmed at 18.3 mL·s^−1^ (i.e., 55 mL·puff^−1^) and the flow profile was square (as required in the same standard).

In [25], the number of series (defined in the AFNOR XP-D90-300-3 standard [24]) was proven to have no influence on the MVE. Based on this observation, our experiments were only composed of 40 puffs divided in 2 series of 20 puffs and each series was separated by a 5-min-break referred to as inter-series. The clearomisers inclination was set at 45° during the vaporization process and back to 0° with respect to the vertical position, during 10 s, between two series in order to simulate the user’s behavior as defined in the AFNOR standard. In each commercial analysis, emissions generations are realized once whereas in our study, each performed experiment was in triplicate (three measurements for each of the tested e-liquids). The densities measured three times, the associated standard deviations, the theoretical ones calculated using Equation (1), and the standard deviations between measured and theoretical densities are reported in Table 5.

The measured densities are repeatable and have a low deviation according to the evaluated ones.

## 3. Results

### 3.1. Characterization of the 20 Reference Liquids

#### 3.1.1. Characterization of the Studied Mixtures

**Figure 4:**
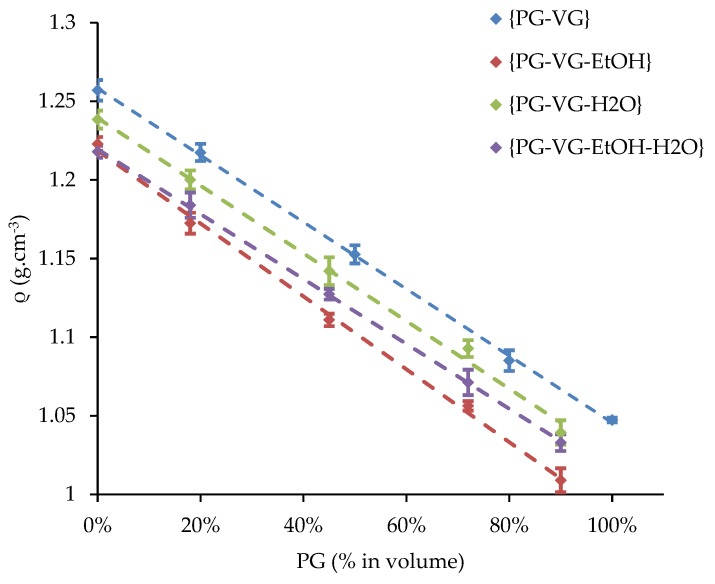
Influence of the PG volume fraction of {PG-VG}, {PG-VG-EtOH}, {PG-VG-H_2_O}, and {PG-VG-EtOH-H_2_O} mixtures on their densities (measured at ambient temperature).

**Figure 5:**
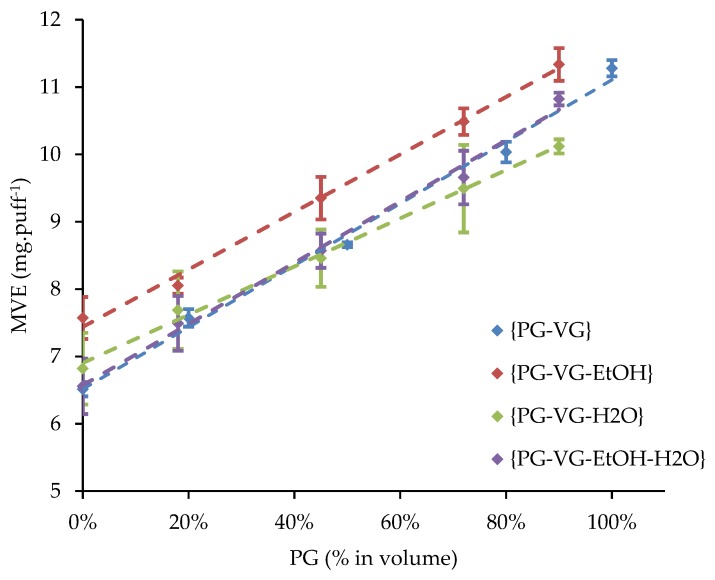
Influence of the PG volume fraction of {PG-VG}, {PG-VG-EtOH}, {PG-VG-H_2_O}, and {PG-VG-EtOH-H_2_O} mixtures on their MVE (measured at ambient temperature, with a supplied power of 15 W).

**Figure 6:**
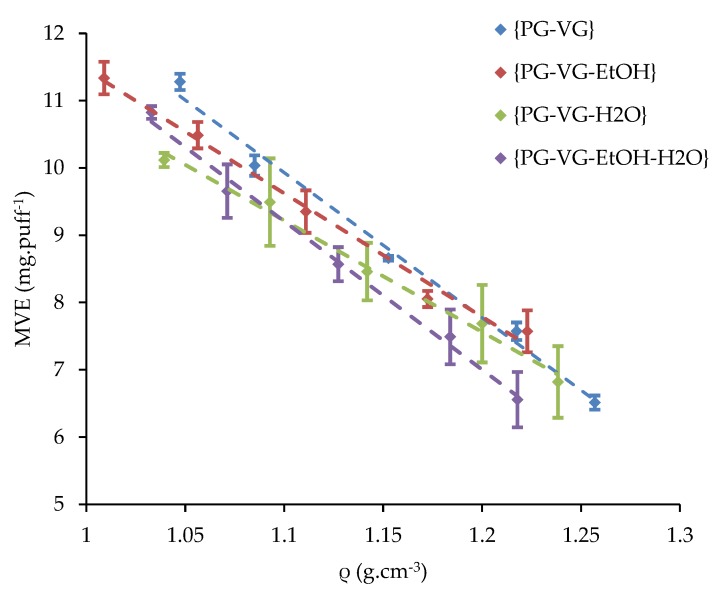
Influence of the densities of {PG-VG}, {PG-VG-EtOH}, {PG-VG-H_2_O}, and {PG-VG-EtOH-H_2_O} mixtures on their MVE (measured at ambient temperature, with a supplied power of 15 W).

The density of each of the 20 liquids was previously measured at ambient temperature and compared to a linear predictive model. The influence of the PG volume fraction of {PG-VG}, {PG-VG-EtOH}, {PG-VG-H_2_O}, and {PG-VG-EtOH-H_2_O} mixtures on their e-liquid is illustrated in Figure 4. The MVE, measured at ambient temperature and obtained with a supplied power of 15 W versus the measured density as well as versus the PG volume fraction were also plotted for all the studied mixtures (see Figure 5 and Figure 6).

As can be seen in Figure 6, all the curves of MVE versus densities of all the studied mixtures are linear. This linearity between MVE and density (ρ) is expressed thereafter using Equation (2):

(2) MVE=aρ+b,

with a (slope) and b (intercept) reported in Table 6 for the four ranges of mixtures.

The obtained results are discussed for all the studied binary, ternary and quaternary systems hereafter.

The composition of the {PG-VG} binary mixtures made its density vary, which influenced the MVE. Indeed, the higher VG volume fraction (respectively, PG volume fraction), the higher (respectively lower) the density of the {PG-VG} mixture was. Besides, the MVE decreased linearly with an increase in density (see Figure 4). The heat capacities and enthalpies of vaporization of PG and VG were close (see Table 4) so the averages of the required energies to increase the temperature up to vaporization of a mixture of these two compounds were not significantly affected. Indeed, the wire heated to the boiling point in a shorter heating duration when PG was used than VG and then, the e-liquid vaporization began. During the rest of the puff duration, the e-liquid was vaporized. At the end, the vaporization duration was longer using PG, leading to a higher MVE. The PG–VG linear fit was used as a reference in Figure 6 in order to characterize the addition of EtOH and/or H_2_O.

In total, 10% of the EtOH volume fraction was added to the previous {PG-VG} mixtures. For the same {PG-VG} volume fractions, we can observe in Figure 6 a shift in the dots that results in an increase in MVE with a decrease of the density values. The fit was linear as for PG–VG trend line. Like for PG and VG, the EtOH heat capacity and enthalpy of vaporization were also close to PG and VG (see Table 4). The slope and the intercept for the EtOH range are reported in Table 6. We can observe a small gap between the slope of {PG-VG} and the range with EtOH that changed from −20.24 to −18.85 × 10^−3^ cm^3^·puff^−1^. The {PG-VG} dots were lower than the {PG-VG-EtOH (10%)} ones.

Furthermore, 10% of the H_2_O range was added to the {PG-VG} mixtures, and the obtained fit was linear as for the {PG-VG} mixtures (see Figure 6). The slope and the intercept for the 10% volume fraction are reported in Table 6. We can observe an important gap between the slopes obtained for {PG-VG} without and with the range of H_2_O that changed from −20.24 to −15.78 × 10^−3^ cm^3^·puff^−1^. The {PG-VG-H_2_O} dots had less repeatability than the {PG-VG} and {PG-VG-EtOH} ones. For the same PG-VG ratio, the {PG-VG-H_2_O (10%)} dots were lower than the {PG-VG} ones mostly at high PG volume fractions (see Figure 5). In contrast to EtOH, the heat capacity and enthalpy of vaporization of H_2_O were close to twice the PG and VG ones (see Table 4). Although, the H_2_O boiling point was close to half of the PG and a third of the VG ones, the required energy to increase in temperature and then to vaporize a liquid that contains H_2_O will be higher than without.

In total, 5% of H_2_O and 5% of EtOH were added to the {PG-VG} mixtures. Its fit was also linear. The {PG-VG} and {PG-VG-EtOH-H_2_O (5–5%)} fits seemed to be overloaded. Their slopes and intercepts were, respectively, 1.3% and 3.2% deviations. Due to the azeotrope {EtOH-H_2_O}, a deeper investigation could not be conducted until its influence on the quaternary system {PG-VG-EtOH-H_2_O} was studied.

Figure 6 also illustrates the MVE versus their densities for the quaternary liquids. The fit was, like the {PG-VG} trend line, linear.

#### 3.1.2. All Liquids with EtOH and/or H_2_O at Volume Concentrations Lower than 10%

**Figure 7:**
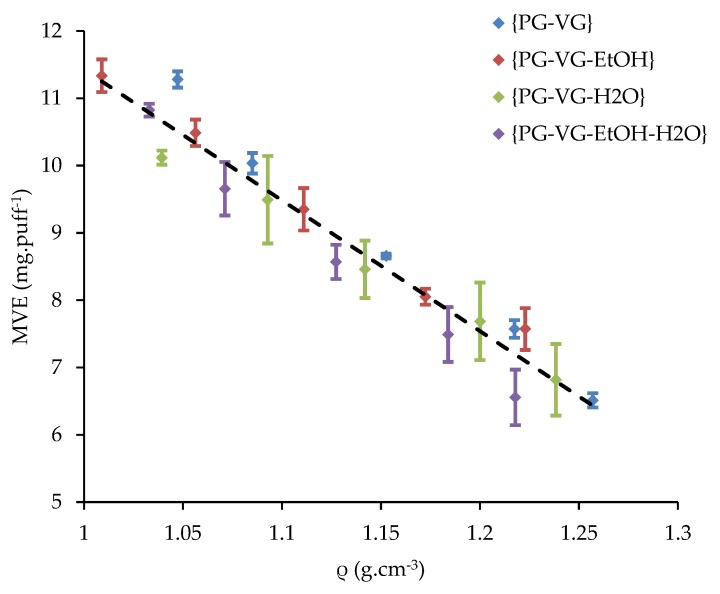
MVE—Influence of the density using {PG-VG}, {PG-VG-EtOH (10%)}, {PG-VG-H_2_O (10%)}, and {PG-VG-EtOH-H_2_O (5%–5%)} ranges.

In Figure 7, the four ranges are overlapped. All the dots seem to follow the same trend of the higher observed dispersion.

The four mixed ranges’ R^2^ value was lower than the ones of each previous studied range that were above than 0.99. Table 7 reports the slopes and the intercepts for the four ranges. The dispersion, confirmed by the R^2^, indicates that a global relation linking MVE and density is not simple and evident to obtain.

Based on this, an experimental method for MVE prediction using its density is proposed. The evaluation of this was done with the reduced linear Equation (3):

(3) MVE=−(18.63±1.00)ρ+(29.86±1.14).

This relation is used in the following sections to verify the 800 commercial analyses.

### 3.2. Characterization of 800 Commercial E-Liquids

#### 3.2.1. The 642 E-Liquids with Known Compositions

**Figure 8:**
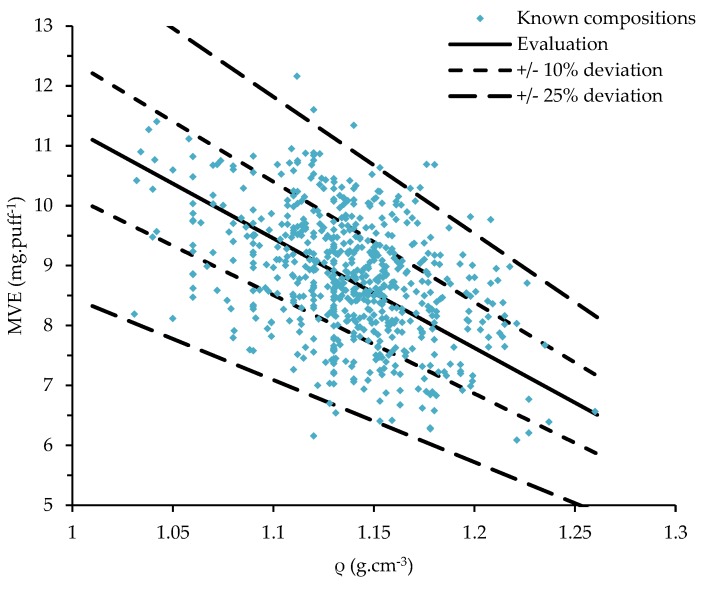
MVE—Distribution of the measured values for the liquids with known compositions.

The 642 commercial liquids with known compositions were tested. These measured densities were used with Equation (2) to evaluate the MVE after the experimentation. The results are reported in Figure 8 as well as the calibrated values, and the ±10% and ±25% of deviations. In total, 97.8% of the measurements were in the ±25%, 61.2% in the ±10%, and 6.9% in the ±1%.

#### 3.2.2. The 158 Liquids of Unknown Composition

**Figure 9:**
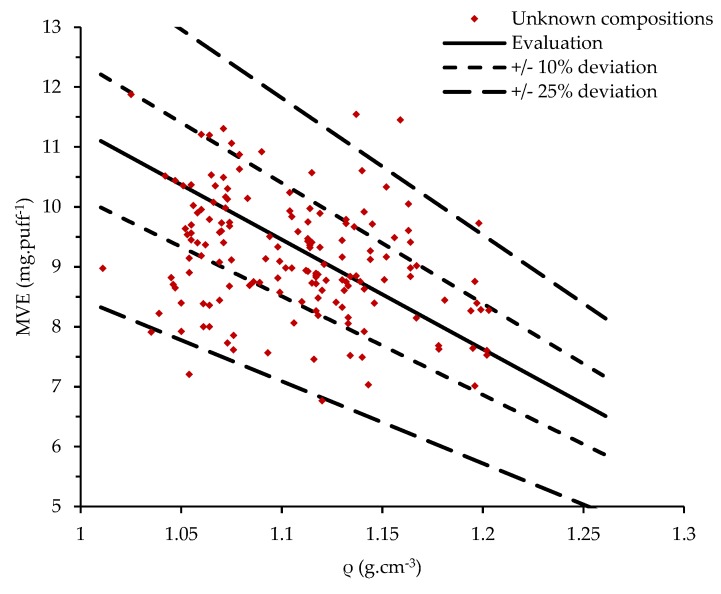
MVE—Distribution of the measured values for the liquids with unknown compositions.

In total, 158 liquids of unknown compositions were also tested using Equation (3). The results are reported in Figure 9 as well as the calibrated values, and the ±10% and ±25% of deviations. In total, 96.8% of the measurements were in the ±25%, 64.6% in the ± 10%, and 9.5% in the ±1%.

## 4. Discussion

**Figure 10:**
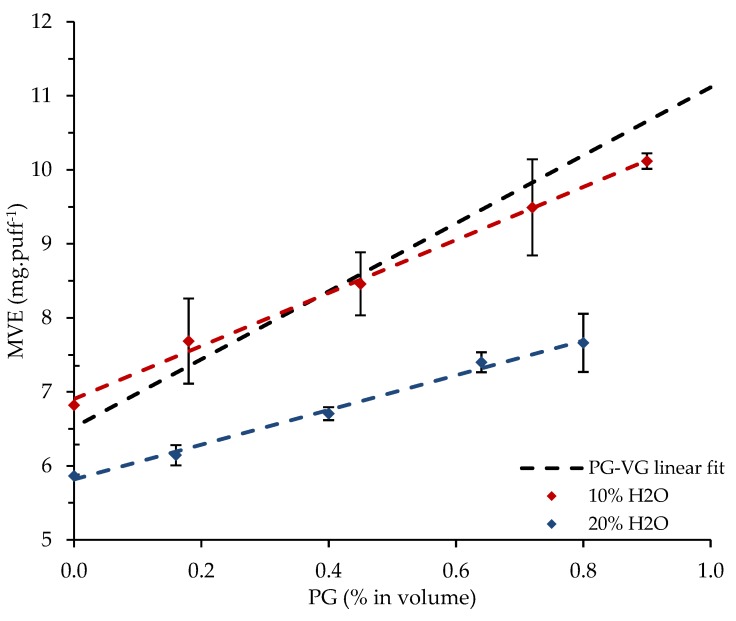
Influence of the PG volume fraction of {PG-VG}, {PG-VG-H_2_O (10%)}, and {PG-VG-H_2_O (20%)} mixtures on their MVE (measured at ambient temperature).

The results highlight that:

- Liquids with a high ratio of PG are vaporized in the highest quantity compared to the ones with a high ratio of VG. PG has a boiling point of 460.8 K and VG of 561.0 K. This means that for the same puff duration (3 s) and the same supplied power (15 W), as the resistance is acting as a heating ramp, the VG boiling point will be reached later than PG one. Besides, as PG and VG have comparable enthalpies of vaporization (respectively, 874 J/g for PG and 996 J/g for VG), the higher the VG ratio in the mixture is, the lower the MVE will be.
- At the same PG ratio, for liquids containing EtOH, the MVE is higher than those containing H_2_O. Yet, ethanol and water have quite close boiling points (respectively, 351.4 K for EtOH and 373.2 K for H_2_O) compared to PG and VG. Ethanol has a vaporization enthalpy of 919 J/g between PG and VG ones. The heat capacity and enthalpy of vaporization of H_2_O are twice the one of PG or VG. Therefore, H_2_O requires more energy than EtOH to heat and vaporize the same mass of liquid. When both EtOH and H_2_O are added in e-liquid, no conclusion could be reached with this work. A thermodynamical study allowing an understanding the behavior of the quaternary systems in e-liquid should be conducted. Additional analyses were conducted in order to gain deeper insight in the characterization of the H_2_O addition on e-liquid. A non-representative range of {PG-VG-H_2_O} systems with 20% of H_2_O were created and vaporized following the described protocol. Figure 10 shows the influence of H_2_O in the e-liquid and the ternary systems {PG-VG-H_2_O} with 10% and 20% are reported. The {PG-VG-H_2_O (20%)} dots are below the {PG-VG-H_2_O (10%)} and {PG-VG} one, revealing an important reduction in the vaporization efficiency for the same energy supplied due to H_2_O addition.

The method was extrapolated from a linear-fit applied on the five ranges tested with EtOH and/or H_2_O concentrations lower than 10%. With this approach, 781 MVE measured were in the range of the previously mentioned 25% of deviation (495 on the 10%) according to the calculated value. A multi-linear method could improve the results but was not tested in these experiments. The linear approach gives sufficient results and deeper analyses were not conducted for now. A main study on the variability of the coil should be firstly realized in order to evaluate the dispersion in the results according to the same device. Furthermore, the 19 other analyses out of the method were not done again because they were realized before the development of this method and the coils were destroyed.

The experimental method presented is only available for the Cubis 1 Ω coil with a supplied power of 15 W and is used to check the emission generation for TPD compliance at LFEL. As there are no standardized devices for emission generation, the methodology proposed here could be applied in other laboratories realizing emission generation. With a correct calibration of the used devices, the vaporization process could be checked rapidly and thus the production of unrealistic results could be avoided in emission generation analyses. Any change in the protocol will require a new calibration.

The approach is based on the consideration that an e-liquid could be approached to a mixture of PG, VG EtOH, and H_2_O. For the e-liquids with a known composition, a deeper analysis might be considered in order to search for other molecules that could be present at significant concentrations.

## 5. Conclusions

This paper focused on the impact of e-liquid composition (PG:VG ratio, ethanol, and/or water adding) on the MVE. The first series of experiments consisted in studying the influence of the supplied power on the four pure liquids (PG, VG, EtOH, and H_2_O) using the Cubis 1 Ω clearomiser in its power range, i.e., from 10 to 25 W. The emission protocol was realized according to the AFNOR standard but reduced at 2 series of 20 puffs instead of 5. Emissions were generated with a U-SAV vaping machine. Then, the influence of the e-liquid composition was tested at a fixed and determined power of 15 W and for one inhalation profile (3 s puff duration and 55 mLof puff volume). In total, 20 reference liquids were designed separately in a binary ({PG-VG}), two ternary ({PG-VG-EtOH} and {PG-VG-H_2_O}), and a quaternary ({PG-VG-EtOH-H_2_O}) system. These series showed that ethanol has a low boiling point, heat capacity, and enthalpy of vaporization, and was vaporized easier than the three other pure liquids and its addition in e-liquid increased the mass of e-liquid vaporized. In opposition, water was found to have a high heat capacity and enthalpy of vaporization despite its low boiling point, which made its vaporization more expensive energetically and reduced the mass of e-liquid vaporized. Finally, PG was vaporized in a higher quantity than VG due to its lower boiling point. Furthermore, the higher supplied powers are, the shorter the times required to reach the boiling point of PG and VG are. Their heat capacities and enthalpies of vaporization were found to be close, leading to a convergence in their MVE at high power.

Then, the reference liquids were used as a calibration step and a linear relation was extracted. It gave a prediction of the MVE after a standardized emission generation for Tobacco Product Directive notifications. In total, 800 commercial liquids were analyzed. The MVE values were compared to the predicted value. It was found that 98% of the 800 liquids gave results presenting deviations lower than ±25% according to the calculated value. Furthermore, 62% had an absolute deviation lower than ±10%. The proposed methodology showed good results. In conclusion, a simple calibration method for emission generation could be developed as:

1. Identification of the optimal power regimes for the PG, VG, and EtOH vaporization for each reference clearomiser used for emission generation.
2. Calibration process using as many reference systems of PG-VG as possible and measurement of their densities.
3. Measurement of the masses of the vaporized liquids.
4. Establishment of a relation linking the e-liquid tested using the selected protocol to the MVE from the results given from the reference systems.
5. Finally, the emission generation of any commercial liquid could be tested and checked only by measuring its density before the experiments.
6. Testing of any emission generation of any commercial liquid and its validation only by measuring the liquid density before the experiments.

As previously explained, the presented results were obtained using a specific protocol. In a previous publication, we proved that the MEV increases with the power supplied in the optimal regime. Any power change will give different results and the calibration will have to be redone. Furthermore, the inhalation profile would have an impact on the MEV. Indeed, the standardized protocol used is based on a low inhalation profile. The inhaled volume (55 mL) corresponds to a volume close to the mouth one and is adapted for mouth-to-lung characterization. With an intense profile of inhalation characteristic of direct-to-lung inhalation, the volume inhaled is closer to the lung volume (tidal volume = 500 mL). This leads to the airflow being extensively increased and might give completely different results with devices that generate important quantities of vapor. Consequently, after the study of the device in terms of supplied powers and of the e-liquids in terms of composition and densities, further works will consist of studying the e-cigarette user’s influence regarding their inhalation profile. These future results combined with the present and previously presented ones will allow an understanding of the influence of the main involved physical quantities to be obtained, allowing the improvement of e-cig’s performances.

## Figures and Tables

**Table 1:** List of the studied pure liquids and general information about them.

Materials	Acronym	CAS Number	Formula	Provider	Purity
Purified water	H_2_O	7732-18-5	H_2_O		
Anhydrous ethanol	EtOH	64-17-5	C_2_H_6_O	GROSSERON	30 ppm of water
1,2-propanediol	PG	57-55-6	C_3_H_8_O_2_	BRENNTAG	≥99.8%
1,2,3-propanetriol	VG	56-81-5	C_3_H_8_O_3_	AMI CHIMIE	99.5%

**Table 2:** Properties of the studied liquids given at ambient temperature (Design Institute for Physical Properties (DIPPR) database: https://www.aiche.org/dippr).

Acronym	M (g·mol^−1^)	ρ (g·cm^−3^)	T_b_ (K)	λ (W·m^−1^·K^−1^)	C_p_ (J·g^−1^·K^−1^)	H_v_ (J·g^−1^)
H_2_O	18.02	0.997	373.2	0.61	4.18	2256
EtOH	46.07	0.790	351.4	0.17	2.44	919
PG	76.09	1.036	460.8	0.15	2.50	874
VG	92.09	1.261	561.0	0.29	2.26	996

**Table 3:** Manufacturer’s general information about the reference clearomiser in this study (Cubis clearomiser with a 1 Ω coil).

Manufacturer	Reference	Resistance	Metal	Wick	Notation	Min	Max
Joytech	Cubis	1 Ω	SS316L	Organic cotton	Cub1	10 W	25 W

**Table 4:** MVE (mass of vaporized e-liquids) obtained with both installations at 15 W, the classical one (with traps) and the specific one (without trap) using pure PG and pure VG representing both extremities of the studied e-liquids’ density range.

Pure Liquids	Specific Installation		Classic Installation	
	MVE (mg·puff^−1^)	Standard Deviation (mg·puff^−1^)	MVE (mg·puff^−1^)	Standard Deviation (mg·puff^−1^)
100% PG	11.28	0.12	11.05	0.71
100% VG	6.51	0.11	6.77	0.51

**Table 5:** Composition, measured density (ρ_m_) and standard deviations (Std_m_) over the triplicate, theoretical densities (ρ_ev_) (Equation (1)) and relative deviations (Rd) between the measured and theoretical densities of the tested PG–VG binary systems at 20 °C.

Volume Percent (%)				ρ_m_ (g·cm^−3^)	Std_m_ (g·cm^−3^)	ρ_ev_ (g·cm^−3^)	Rd (%)
EtOH	H_2_O	PG	VG				
0	0	0	100	1.2570	0.0016	1.2613	0.34
0	0	20	80	1.2174	0.0066	1.2165	0.07
0	0	50	50	1.1526	0.0057	1.1490	0.31
0	0	80	20	1.0851	0.0055	1.0816	0.32
0	0	100	0	1.0473	0.0065	1.0364	1.05
10	0	0	90	1.2229	0.0044	1.2306	0.63
10	0	18	72	1.1725	0.0067	1.1936	1.77
10	0	45	45	1.1110	0.0040	1.1329	1.93
10	0	72	18	1.0563	0.0030	1.0651	0.83
10	0	90	0	1.0090	0.0076	1.0153	0.62
0	10	0	90	1.2384	0.0057	1.2399	0.12
0	10	18	72	1.2000	0.0060	1.2038	0.32
0	10	45	45	1.1419	0.0088	1.1448	0.25
0	10	72	18	1.0928	0.0054	1.0790	1.28
0	10	90	0	1.0393	0.0078	1.0307	0.83
5	5	0	90	1.2179	0.0039	1.2353	1.41
5	5	18	72	1.1839	0.0081	1.1987	1.23
5	5	45	45	1.1273	0.0034	1.1389	1.02
5	5	72	18	1.0712	0.0081	1.0721	0.08
5	5	90	0	1.0329	0.0053	1.0231	0.96

**Table 6:** MVE—Values of the coefficients, a and b, and their standard deviations, Δa and Δb, in Equation (2) of the {PG-VG}, {PG-VG-EtOH}, {PG-VG-H_2_O}, and {PG-VG-EtOH-H_2_O} ranges (R^2^ = coefficient of determination).

Volume Percent (%)				a (x10^−3^.cm^3^.puff^−1^)	Δa (x10^−3^.cm^3^.puff^−1^)	b (mg·puff^−1^)	Δb (mg·puff^−1^)	R^2^
EtOH	H_2_O	PG	VG					
0	0	0	100	−20.24	0.94	32.05	1.08	0.9915
0	0	20	80					
0	0	50	50					
0	0	80	20					
0	0	100	0					
10	0	0	90	−18.85	0.74	30.32	0.83	0.9938
10	0	18	72					
10	0	45	45					
10	0	72	18					
10	0	90	0					
0	10	0	100	−15.78	0.58	26.39	0.66	0.9946
0	10	20	80					
0	10	50	50					
0	10	80	20					
0	10	100	0					
5	5	0	90	−19.97	0.93	31.03	1.05	0.9913
5	5	18	72					
5	5	45	45					
5	5	72	18					
5	5	90	0					

**Table 7:** MVE—Values of the coefficients (a) and (b) and their standard deviations (Δa) and (Δb) in Equation (2) of the {PG-VG}, {PG-VG-EtOH}, {PG-VG-H_2_O}, and {PG-VG-EtOH-H_2_O} ranges (R^2^ = coefficient of determination).

	a (×10^−3^ cm^3^·puff^−1^)	Δa (×10^−3^ cm^3^·puff^−1^)	b (mg·puff^−1^)	Δb (mg·puff^−1^)	R^2^
4 ranges	−18.63	1.00	29.86	1.14	0.9475
